# Subtle changes in perivascular endometrial mesenchymal stem cells after local endometrial injury in recurrent implantation failure

**DOI:** 10.1038/s41598-023-27388-8

**Published:** 2023-01-05

**Authors:** Yiping Fan, Ryan Wai Kheong Lee, Xiang Wen Ng, Caroline E. Gargett, Jerry Kok Yen Chan

**Affiliations:** 1 Department of Reproductive Medicine, KK Women’s and Children’s Hospital, Singapore, Singapore; 2 Experimental Fetal Medicine Group, Department of Obstetrics and Gynaecology, Yong Loo Lin School of Medicine, National University Health System, Singapore, Singapore; 3 Academic Clinical Program in Obstetrics and Gynaecology, Duke-NUS Medical School, Singapore, Singapore; 4 Department of Maternal Fetal Medicine, KK Women’s and Children’s Hospital, Singapore, Singapore; 5 The Ritchie Centre, Hudson Institute of Medical Research, Clayton, VIC Australia; 6 Department of Obstetrics and Gynaecology, Monash University, Clayton, VIC Australia

**Keywords:** Developmental biology, Stem cells, Medical research

## Abstract

Improvements in reproductive techniques have resulted in the live birth rates from IVF procedures increasing from 5% to approximately 30% in recent decades but has plateaued since. Emerging preclinical and clinical data implicates endometrial receptivity deficiencies in patients with recurrent implantation failure (RIF) as the predominant factor hindering successful implantation. Mechanisms on how local endometrial injury (LEI) improves implantation rates in patients with RIF are currently unknown. We hypothesized that LEI may influence perivascular endometrial mesenchymal stem/progenitor cells (eMSCs) which are thought to regenerate the stromal vascular component of the functional layer every month. Here, we assessed the effect of LEI on the proportion and function of eMSCs present in consecutive LEI biopsies. Consecutive paired mid-luteal phase endometrial biopsies obtained from patients with RIF were digested to single cells and the proportion of SUSD2-expressing cells determined. Growth kinetics and decidualization were compared between the consecutive LEI samples. A mid-luteal LEI altered the decidualization capacity of SUSD2^+^ eMSCs in women with RIF, but not their proportion or clonogenicity. With the potential of LEI to improve IVF outcomes in women with RIF, additional investigations are needed to understand the impact of the altered decidualization response in eMSCs.

## Introduction

In vitro fertilization (IVF) has allowed 30% of couples facing infertility to successfully achieve viable pregnancies^1^. However, there are patients who repeatedly fail to fall pregnant despite transfers of good quality embryos. Recurrent implantation failure (RIF) in our hospital setting refers to cases where women failed to achieve a clinical pregnancy after a transfer of four good-quality embryos in a minimum of two IVF cycles. Until recently, one of the most often used adjunct therapies in IVF was to generate a local endometrial injury (LEI) known as endometrial scratching, in an attempt to improve success rates^2^. In fact, eighty-three percent of clinicians from United Kingdom, Australia, and New Zealand recommend LEI in a survey conducted as recently as 2015^3^.

Since the initial case report in 2003, multiple trials of LEI have been conducted which generally only showed benefit in those with RIF^4–7^, but not in other unselected populations^8^. Using a retrospective study, Kitaya et al. however found no difference in the clinical pregnancy rate following LEI in women with RIF except where there is a co-morbidity, for instance polycystic ovarian syndrome^9^. The lack of high quality randomized controlled trials (RCTs)^10^, the different definitions of RIF and the varied types of intervention led to the use of meta-analyses to guide clinical practice^10^. A meta-analysis by Vitagliano et al. in 2018 showed possible benefits of LEI for those with two or more failed embryo-transfer (ET) cycles, particularly where a double-luteal phase LEI had been performed^11^. More recently, high quality RCTs have shown opposing findings, albeit with different patient groups and LEI intervention protocols^6,12,13^.

The proposed mechanism of action with LEI ranges from mechanical disruption to the endometrium correcting the asynchrony between endometrial and embryo stages^14^, with a wound healing response possibly influencing immune cell perturbations and activation states favoring implantation^15–17^ and enhanced decidualization^18,19^. Another theory is that LEI induces an inflammatory reaction involving cytokines, macrophages and other immune cells^20^. However, none adequately explain how LEI in a cycle prior to embryo transfer promotes implantation leading to pregnancy and live birth. We propose that endometrial injury activates endometrial stem/progenitor cells in the basal layer to proliferate and provide replacement cells, resulting in the production of a more cellular and thicker functional layer in the subsequent cycle, a concept that can explain carryover effect of LEI in improving endometrial receptivity in the subsequent cycle, given that the basal layer remains during menstruation.

Endometrial stem/progenitor cells were first reported as clonogenic epithelial and stromal cells^21^, which could self-renew in vitro by serial cloning, had high proliferative activity and differentiated into large gland-like structures in 3D culture and mesodermal lineages, respectively^22^. Subsequently, specific markers of clonogenic endometrial mesenchymal stem/progenitor cells (eMSCs) which fulfil the International Society for Cellular Therapy (ISCT) MSC criteria were identified showing that eMSCs were located around blood vessels^23^. This was confirmed by a single perivascular marker, Sushi domain containing-2 (SUSD2) (identified by the W5C5 antibody) which also enriched for clonogenic eMSCs and is particularly successful in selecting endometrial mesenchymal stem cells from endometrial tissues^24^ and body fluids^25^. SUSD2^+^ cells comprised 4.2% of the freshly sorted endometrial stromal cells which differentiated into adipocytes, osteocytes, chondrocytes, myocytes, and endothelial cells, and generated endometrial stromal-like tissue in vivo. These eMSCs are also reduced in obese women with a history of reproductive failure, indicative of a role they play in fertility^26^.

Specific molecular and cellular defects in the endometrium could account for implantation failure^27^ and that forms the basis of our study that eMSCs likely mediate cyclic regeneration of the stromal/vascular component of the endometrial functional layer. SUSD2^+^ eMSCs constitute a dynamic population of cells enabling the endometrium to adapt to local injury by fulfilling their stem cell role in stromal vascular tissue regeneration. Therefore, we aimed to investigate the impact of LEI on eMSCs and determine if LEI alters their proportion and capacity for decidualization, which in turn affects endometrial tissue remodeling explaining the improved rates of pregnancy outcomes post LEI. In this study, we sought to determine perturbations in the perivascular eMSC compartment after a luteal phase LEI in women with RIF. We leveraged on a sequential mid-luteal phase LEI protocol to compare the baseline biopsy with a subsequent biopsy where an implantation event would be expected in single luteal LEI protocol.

## Results

A total of 32 patients were recruited and paired LEI samples were obtained from 26 patients. Participant’s age ranged from 28 to 41 years (Table 1, details in Supplementary Table 1). All patients recruited had failed to achieve a clinical pregnancy after a transfer of four or more top-quality cleavage-stage embryos or two or more top-quality blastocysts in a minimum of two previous IVF embryo-transfer cycles except for patient 1 (Supplementary Table 1). The sample for patient 1 was used for the frequency of SUSD2^+^ cells and the clonogenicity experiments. Removal of the data from patient I from the analyses did not impact on the outcome. Two patients dropped out after LEI, before their embryo transfers. After the second LEI, 14 out of 24 (58.3%) have successful implantation, which resulted in 5 biochemical pregnancies, 0 miscarriage and 9 live births.

**Table 1:** Characteristics of 26 Patients undergoing sequential LEI.

Variable	1st LEI
Age	35.3 ± 3.2
Parity	0.3 ± 0.6
**Type of subfertility**	
Primary	21 (80.8%)
Secondary	5 (19.2%)
**Causes of subfertility**	
Male	11 (42.3%)
Tubal	5 (19.3%)
Diminished ovarian reserve	3 (11.5%)
Anovulation	3 (11.5%)
Endometriosis	3 (11.5%)
Unexplained	1 (3.9%)
**Embryo transfers before LEI**	
No. of transfers (avg)	2.2 ± 0.4
No. of embryos transferred (avg)	3.2 ± 1.0
Total no. D2/3 embryos transferred	47 (54.0%)
Total no. blastocysts transferred	40 (46%)
**Embryo transfers after LEI**	
No. of embryos transferred (avg)	1.7 ± 0.09
Total no. D2/3 Embryos Transferred	11 (45.8%)
Total no. Blastocysts Transferred	13 (54.2%)
Clinical Pregnancy Rate (24 patients)*	9 (37.5%)
Biochemical Pregnancy	5 (20.8%)
Miscarriage	0
Live birth	9 (37.5%) (7 singletons and 2 twins)
Drop Out (no ET done)	2 (8.3%)

Data are mean + /− SD of n = 26 except were indicated*.

### Proportion of SUSD2^+^ eMSCs in LEI biopsies

**Figure 1:**
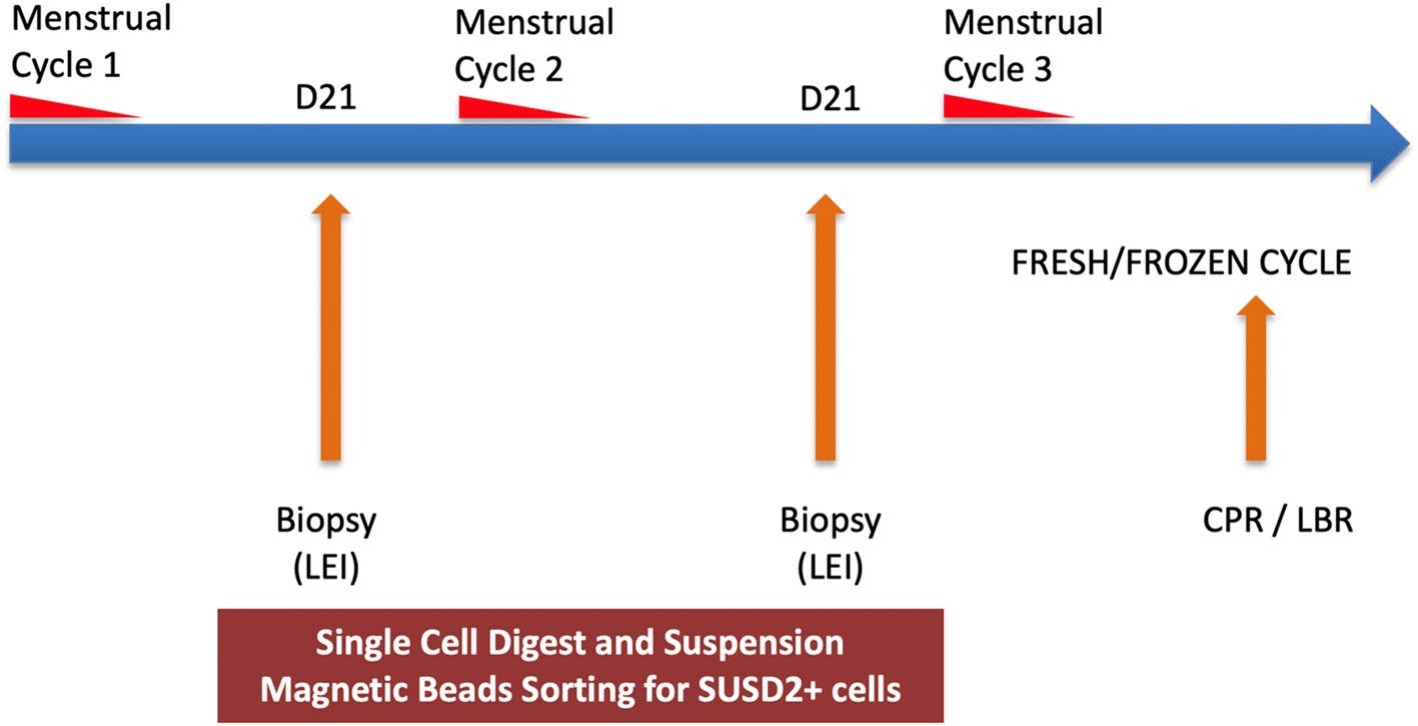
Timeline of endometrial pipelle sampling and experimental analysis. Legend: Local endometrial injury (LEI), Clinical pregnancy rate (CPR), Live birth rate (LBR).

**Figure 2:**
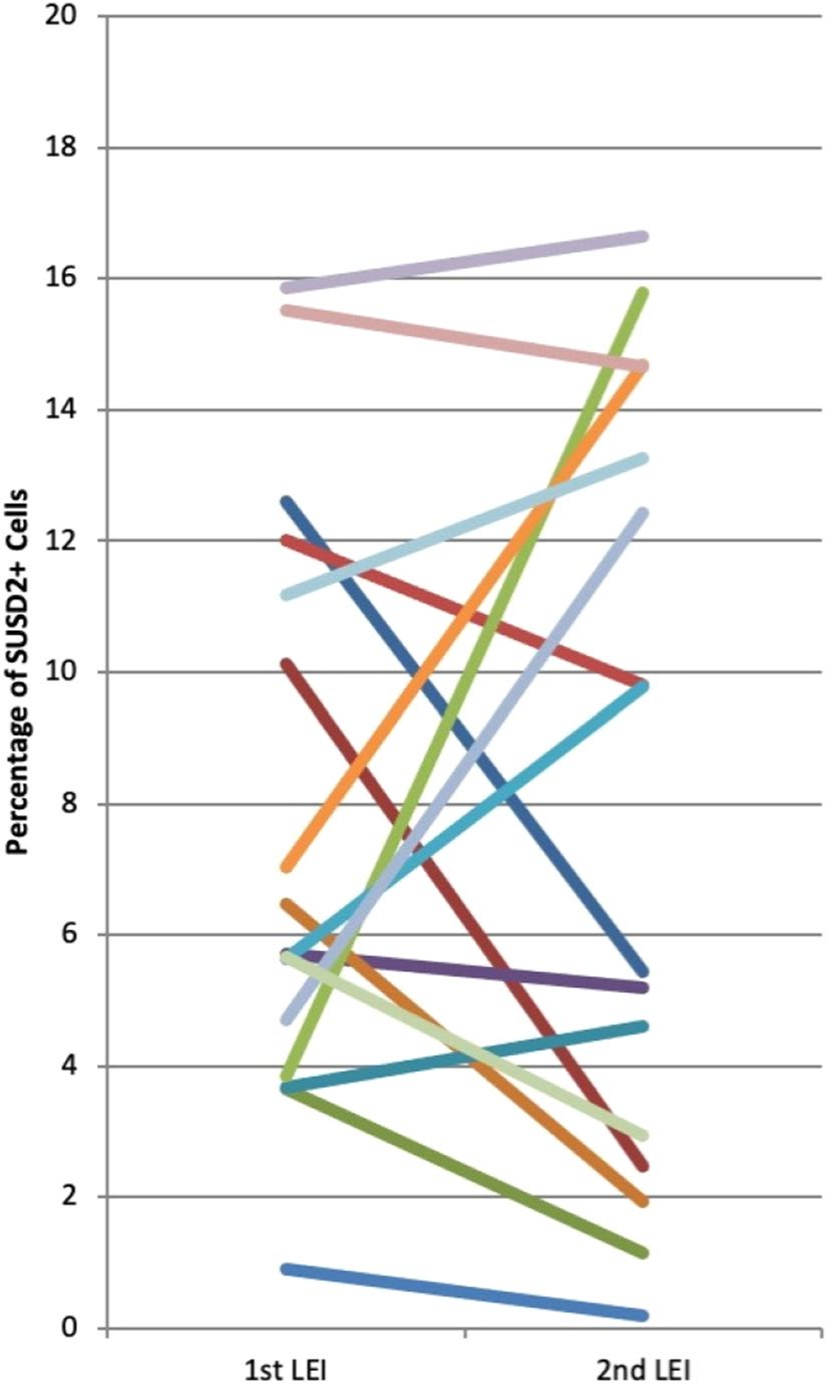
Percentage of SUSD2^+^ cells in freshly prepared stromal cell suspensions of paired samples from the first and second LEI. n = 16.

Using flow cytometry, we compared the proportion of SUSD2^+^ cells in the first and second pipelle biopsy before embryo transfer (Fig. 1). There was no difference in the proportion of SUSD2^+^ cells between the first (median 5.7%, range 0.45–22%, 26 samples) and second LEI sample (median 5.4%, range 0.0–33.9%, 20 samples) (*p* = 0.87 by Mann–Whitney test). Looking specifically at the paired samples (n = 16) where we obtained both first and second LEI from the same patients, there was also no difference in the proportion of SUSD2^+^ cells between the first LEI (median 5.7%, range 0.9–15.9%) and the second LEI (median 5.4%, range 0.19–16.7%) (*p* = 0.82 by Wilcoxon test, Fig. 2).

**Figure 3:**
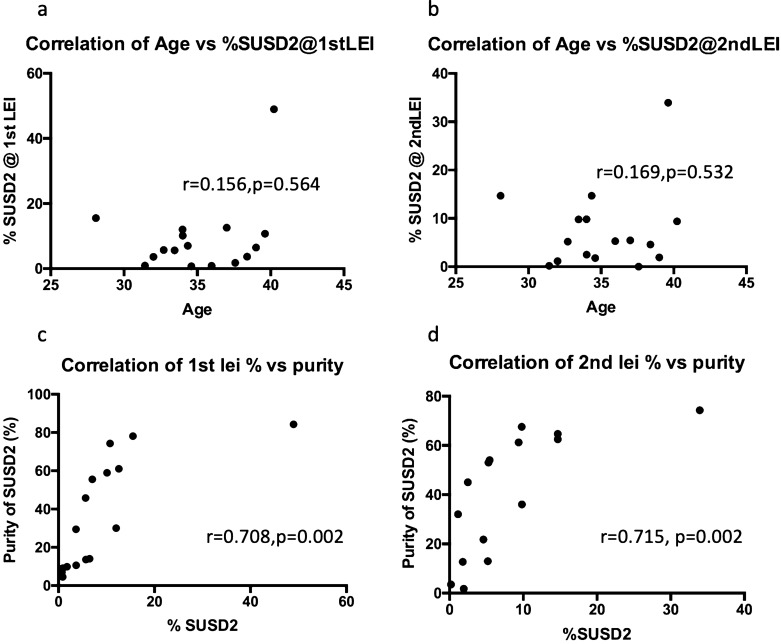
Correlation analysis of %SUSD2^+^ cells and subject age at (**a**) first and (**b**) second LEI and purity following magnetic cell sorting using SUSD2 magnetic beads for first (**c**) and second (**d**) LEI. (n = 26 for first LEI and n = 20 for second LEI).

The proportion of SUSD2^+^ cells was independent of the yield of stromal cells collected from dissociated tissue samples. Neither was there any correlation with the age of the patients (Fig. 3a, b). Magnetic bead sorting did not result in a high purity of SUSD2^+^ cells, with an average of 43% and 49% at the first and second LEI respectively. While the purity of SUSD2^+^ cells did not correlate with the yield of cells dissociated from the tissue, it did correlate with percentage of SUSD2^+^ cells for both first and second LEI (r = 0.708 (*p* = 0.002) and 0.715 (*p* = 0.002), (Fig. 3c, d). Similar to the SUSD2^+^ cells, the proportion of CD45 + leukocytes in the endometrial cell suspensions was comparable between the first and second LEI (15.4 ± 15.4%, range 1.44–55.9%, and 16.0 ± 17.4%, range 1.2–61.6% respectively, *p* = 0.96).

### Clonogenicity and growth kinetics of purified SUSD2^+^ cells

**Figure 4:**
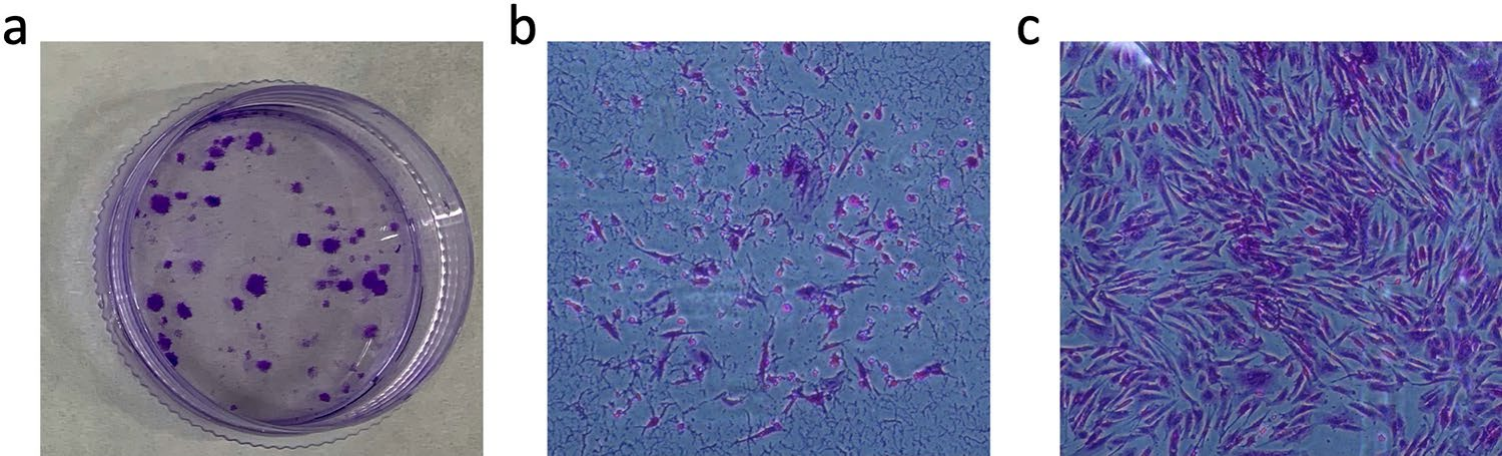
Clonogenicity of magnetic bead sorted SUSD2^+^ cells seeded at a plating density of 200cells/cm^2^. (**a**) Two types of colonies formed after 4 weeks: small colonies of approximately 50 cells loosely packed cells (**b**), and large colonies of more than 200 tightly packed cells (**c**).

Next, we studied the clonogenicity and growth kinetics of the purified SUSD2^+^ cells. We consistently obtained colonies from the SUSD2^+^ population seeded at clonal densities (n = 13 paired samples) (Fig. 4a), generating two distinct types of stromal colonies; small colonies comprising loosely packed cells (Fig. 4b) and large colonies with a dense centre of tightly packed cells (Fig. 4c). Cloning efficiencies were highly variable between samples, although mean values were similar between the first (mean ± SD) 14.7 ± 12.4%, range 1–40%) and second (mean ± SD 19.4 ± 17.8%, range 0–50%) LEI samples (*p* = 0.37, paired Student’s t-test) (Table 2). Doubling times at first passage were 12.9 (median, range 1.9–34.9) days for first LEI and 10.5 (3.5–23.5) days for the second LEI (*p* = 0.79), 5.6 (2.0–29.1) versus 2.7 (1.5–4.6) days at second passage (*p* = 0.03), respectively and 6.9 (1.9–26.7) versus 4.0 (1.7–8.2) days, respectively at third passage (*p* = 0.52) (Table 2) (n = 6 unpaired samples/group). There was an apparent trend towards a shorter doubling time for cultured SUSD2^+^ cells in the second LEI samples with less variation between samples and which is statistically significant at passage two.

**Table 2:** SUSD2^+^ eMSCs Growth Kinetics.

Measurement	1st LEI	2nd LEI	*p* value
Cloning efficiency (%)	14.7 ± 12.4	19.4 ± 17.8	0.37^
Doubling time @ P1 (days)	12.9 (1.9–34.9)	10.49 (3.5–23.5)	0.79^^
Doubling time @ P2 (days)	5.6 (2.0–29.1)	2.7 (1.5–4.6)	0.03^^
Doubling time @ P3 (days)	6.9 (1.9–26.7)	4.0 (1.7–8.2)	0.52^^

Cloning efficiency data are mean ± SD of 6 samples/group, ^paired Student’s t-test.

Doubling time data are median (range) of n = 6 samples/group ^^ Mann–Whitney tests.

### SUSD2^+^ cells decidualize in response to increased cAMP levels

**Figure 5:**
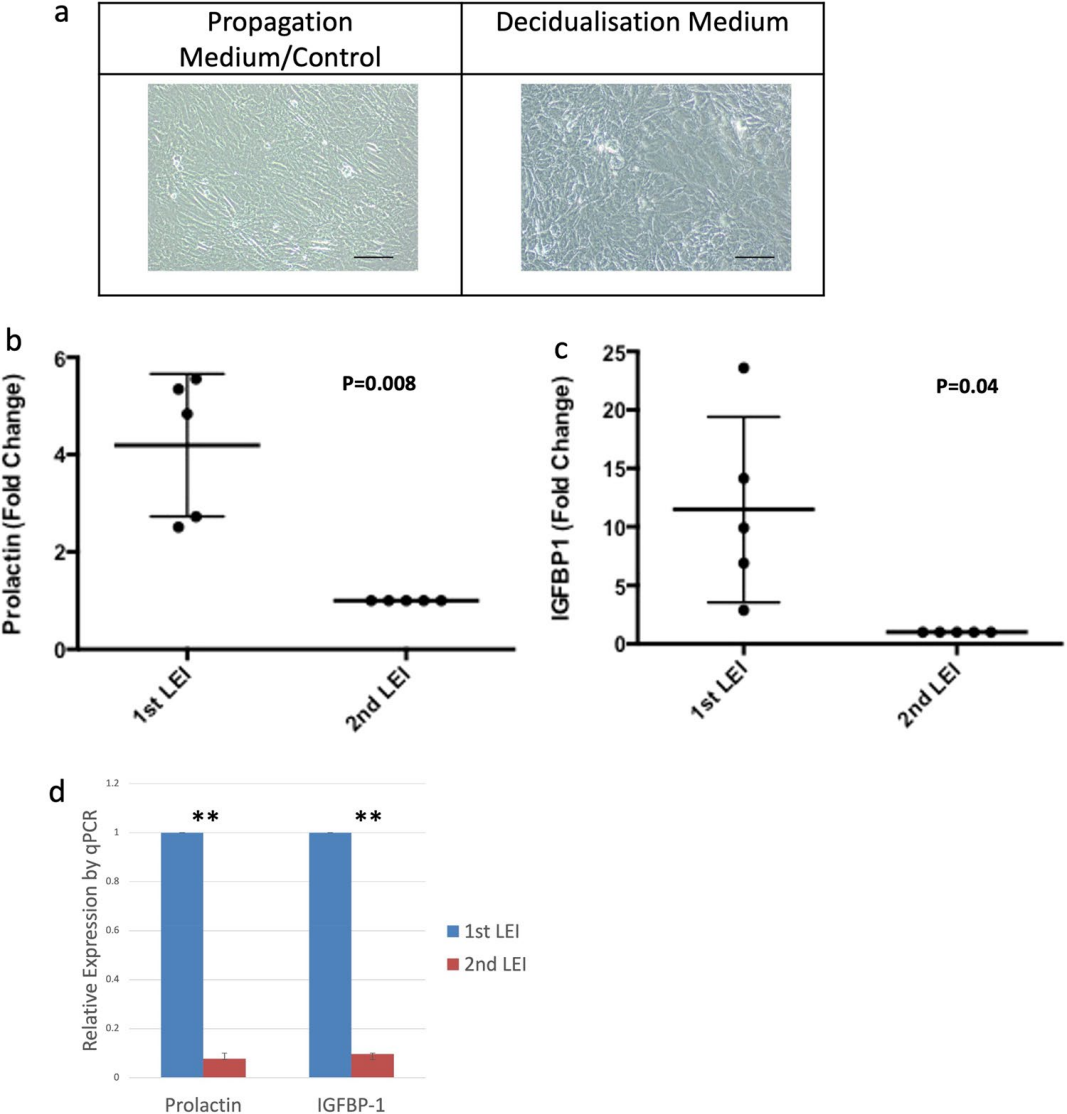
Altered decidualization capacity of SUSD2^+^ cells between first and second LEI. Confluent SUSD2^+^ cells adopt an epitheloid shape with prominent nucleoli and denser cytoplasm in the presence of MPA and 8-bromo-cAMP (**a**). Scale bar 100um. All 5 samples showed a reduction in Prolactin (**b**) and IGFBP1 (**c**) protein levels in the supernatant between first and second LEI. Relative expression of both *PRL* (Prolactin) and *IGFBP1* mRNA by qPCR also showed marked reduction between (**d**). Data normalized to Prolactin and IGFBP1 levels of cells cultured in propagation medium. **p < 0.01.

In order to study the functional capability of SUSD2^+^ cell cultures, we subjected five paired samples to a decidualization medium. Of the five women who provided samples for the ELISA assays, three had live births, one had biochemical pregnancies and one had no implantation event. SUSD2^+^ cells cultured in medium containing cAMP and progesterone changed from an elongated fibroblast-like morphology to a more epitheloid phenotype, with enlarged and rounded nuclei, increased numbers of nucleoli and denser cytoplasm with more secretory granules containing glycogen and lipid droplets (Fig. 5a). Prolactin secretion was 4.2 ± 1.5 fold higher in the first LEI sample compared to the second LEI culture supernatants (*p* = 0.008) (Fig. 5b). There was a similar reduction in another key decidualization marker IGFBP-1, with 11.5 ± 7.9 fold reduction between the first and second LEI culture supernatants (*p* = 0.04) (Fig. 5c). We corroborated this difference in protein concentrations using qPCR and found a marked decrease in mRNA expression of *PRL* and *IGFBP-1* in decidualized SUSD2^+^ cells in the second LEI sample compared to the first (Paired t-test: *p* < 0.001 and *p* = 0.009 respectively) (Fig. 5d).

## Discussion

In this study, we have shown that SUSD2^+^ cells isolated from two sequential LEI biopsies of women with RIF were clonogenic, highly proliferative and could decidualize. Regardless of the controversy surrounding the use of LEI for treating a RIF population^12^, we have for the first time, defined the effect of a mid-luteal phase LEI on the SUSD2^+^ perivascular cell population in the following cycle in the endometrium. Although the clonogenicity and proportion of SUSD2^+^ cells did not change after a LEI, there was a trend towards a higher proliferative state with lower doubling times on the second LEI. In addition, we showed that the degree of SUSD2^+^ eMSCs decidualization was strikingly reduced in the second LEI using a well validated in vitro assay. This suggests that the SUSD2^+^ cells after a LEI may have adopted a more proliferative and less differentiated status. It is possible that the less differentiated SUSD2^+^ eMSCs in the second LEI resulted in a thicker more vascularized stroma to support embryo implantation in women with RIF. How this leads to a supposed beneficial effect such as increased pregnancy and live birth rates is the subject of further investigations.

The proportion of SUSD2^+^ cells in our RIF population (5.7%) is similar to that previously reported in Lucas et al. (7.2%)^28^ as well as by Masuda et al. (4.1%)^24^. Although we did not find any differences in the proportion of the SUSD2^+^ population after the initial LEI, there was a trend towards higher clonogenicity (31% increase), which was similarly observed in a population of women with recurrent miscarriages, a different condition to RIF^28^. There was also an apparent shorter doubling time for cultured SUSD2^+^ cells from the second LEI in all passages interrogated, which was significant for passage 2. In our study, the doubling time was longer than that reported for menstrual blood-derived eMSCs^29^ using an adherence selection purification method, but similar to another group which used CD117 selection and reported 6–16 days for cells from passage 1 to reach 70% confluency^30^. This observation could be due to the differences in the cell phenotype obtained during an inflammatory shedding event, the culture medium used, and the large number of non-SUSD2 selected stromal cells present in menstrual blood^29,30^. Similar to published findings, age had little impact on the abundance of eMSCs^26,31^.

Endometrial decidualization transforms perivascular eMSCs from elongated fibroblastic-like cells into enlarged round epitheloid cells in culture. Decidualization is essential for embryo implantation, trophoblastic invasion, placentation and pregnancy development^32,33^. Endometrial stromal cell and eMSC decidualization can be recapitulated in vitro by exposing cultured cells to progesterone and a cAMP analog. Prolactin and IGFBP1 secretion by cultured eMSCs served as a measure of their hormone responsiveness and capacity to decidualize^34^. Regardless, there is a reduction in markers of decidualization in perivascular eMSCs after an LEI as indicated by the reduction in prolactin and IGFBP1 secretion from eMSCs cultured from an LEI sample obtained in the subsequent cycle compared with the initial LEI. One theory on the mechanism of endometrial scratching is that it enhances decidualization, which is crucial to implantation^10^, although this was not observed in our study. Unlike mice, decidualization in humans occurs independently of pregnancy but is regulated by hormonal changes, particularly increasing progesterone levels. As endometrial scratching is commonly performed in the cycle prior to embryo transfer, it is hard to comprehend how increased decidualization of the previous cycle will benefit a subsequent one, when the decidualized regions are shed in the intervening menstruation^35^. Our data suggests decidualization is affected by LEI in the following cycle, but reduces rather than enhances this essential mechanism for embryo implantation. A second possibility posited is that a lag phase induced by the LEI slows development of an otherwise advanced endometrium, allowing synchrony between the endometrium and implanting blastocyst^10^, but again hard to rationalize biologically. However, our hypothesis that local injury to endometrium caused by a biopsy taken outside the menstrual phase activates quiescent endometrial MSCs around blood vessels in the unshed basal layer is plausible because the effect will manifest in the subsequent cycle and will not be lost during menstruation. Indeed, in other forms of injury such as hemorrhage, the hemopoietic stem cells activate, rapidly proliferate and produce large numbers of mature red blood cells to rapidly restore circulating red blood cell levels. Similarly, if perivascular eMSCs become activated to proliferate and regenerate the vascularized stroma of the functional layer in the following cycle, they may be responsible for producing a more vascular tissue into which an embryo can implant and is conducive to establishing and maintaining a pregnancy. Since we did not find clear evidence that LEI increased eMSCs content of the functional layer in the second LEI biopsy, it could be that other stem/progenitor cell populations may be responsible for the possibly improved IVF outcomes observed in this study. It could be that the clonogenic, self-renewing N-cadherin+ epithelial progenitors identified in the horizontal rhizome-like endometrial glands^36,37^ or SSEA-1 + SOX9+ ^38^ or AXIN2+ ^39^ epithelial cells, all found in the basal layer, may be activated by LEI, as any proliferative activity of these potential progenitors will be carried over into the subsequent cycle.

Despite several RCTs conducted to determine the efficacy of LEI in improving IVF outcomes, a consensus has still not been reached on its efficacy for RIF women^4,6,12,40^. A study by Liu et al. found no increase in implantation, clinical pregnancy or live birth rates after endometrial injury in the cycle preceding IVF embryo transfer in an unselected population of women, regardless of whether the injury took place during the luteal or proliferative phase^2^. An RCT assessing endometrial scratching in 1,364 women having an IVF cycle also showed no difference in live birth rate, although it was not powered to detect a difference for women with RIF^12^. A meta-analysis of 14 RCTs involving 2537 patients corroborated the findings^40^. It is important to note that for the above studies, the selection criteria differed, ranging^41^ from women undergoing their first IVF^2,8^ to those having 2 or more failed full IVF/ICSI cycles^42,43^. RCTs utilizing mid-luteal phase LEI protocols suggest a clinical benefit. Nastri et al. demonstrated a 1.83 (95CI 1.13–2.97) fold increase in live birth rates (LBR)^4^, Olesen showed higher clinical pregnancy rate but not LBR where there had been three or more failed embryo transfers (ET)^6^, and the SCRaTCH test similarly showed a trend towards benefit^13^. Given the trends observed, it remains possible that a mid-luteal phase LEI conducted singularly or sequentially before the next ET may be of benefit. In our small series of women with a mean age of 35.3 years, who had at least two failed ETs comprising at least four cleavage stage or two blastocyst stage embryos, we achieved a 33% (8/24) LBR. Ata et al. had recently highlighted the importance of maternal age on embryo aneuploidy rates, the leading cause for implantation failure^44,45^. Based on the average age of our participants, published data would suggest an euploidy rate of 45–65%^46–50^ with such a difference markedly influencing implantation rates alone. Therefore, until an accurate definition of RIF accounts for age-related aneuploidy rates, subsequent assignment of women with RIF will reduce the risk of over-diagnosis of this condition and advance the field of treatment of RIF^45^.

The strength of our study is the removal of inter-individual variability by using paired samples with each participant providing endometrial tissue during both first and second LEI. Our study is based on the effect of LEI on purified perivascular eMSCs likely responsible for regenerating the stromal vascular component of endometrial tissue each menstrual cycle, in women with RIF. However, while biological and single cell RNA sequencing studies^51^ confirm that SUSD2 is an excellent marker for perivascular eMSCs, a weakness of our study is that magnetic bead sorting lacks efficacy in purifying SUSD2^+^ eMSCs. While we were able to achieve purities as high as 85%, the average purity of the sorted SUSD2^+^ population was 46%, a level which we have previously reported upon^24^. FACS sorting using SUSD2^24,52^ or CD146/PDGFRβ co-expression^23,53^ are more effective in obtaining purer populations of eMSCs^31^.

Endometrial stromal cells are a heterogenous population, comprising active and quiescent MSCs, transit-amplifying cells, mature fibroblasts and also senescent cells. Failure of homeostatic balancing of the different cells could account for aberrant decidualization which could offer an explanation to our observations in women with RIF. Impairment of decidualization is also associated with infertility, recurrent miscarriages and other medical issues and parsing out the molecular details of optimal decidualization could improve success rates in ART as well as form the basis for the potential development of new treatments in reproductive medicine^54^.

While our study did not indicate endometrial injury stimulation of the perivascular eMSCs, we did observe an apparent improvement of pregnancy outcomes (9/24 live births in women with RIF), albeit in small numbers and in a non-randomized manner. More work is required to prove that endometrial injury increases the expression of genes necessary to produce a receptive endometrium enabling implantation, such as endometrial bladder transmembrane l protein, MUC1, crystallin alpha B, APOD, and PLA2 upregulated as reported by Kalma et al.^55^.

This study examined a stem/progenitor cell activation hypothesis to explain the apparent beneficial effect of LEI on birth outcomes in women with RIF. While limited to just the eMSCs, it could be that epithelial progenitors or basal layer glandular epithelial cells play a role in promoting any beneficial effects in improving successful IVF pregnancy outcomes. While the results thus far indicate more investigation is needed to understand the impact of LEI on improving pregnancy rates in IVF, it highlights the importance of looking at the eMSCs in regenerative medicine and other gynaecological disorders, in addition to recurrent implantation failure such as repeated miscarriages and endometriosis^25^.

## Methods

### Ethics approval

The current study was reviewed and approved by the SingHealth Centralized Institutional Review Board (CIRB 2013/215/D). All participants gave written informed consent before participating in the study at KKIVF Centre, KK Women’s and Children’s Hospital. All experimental procedures were performed in accordance with the relevant guidelines and regulations.

### Inclusion and exclusion criteria

Women undergoing IVF treatment with two failed ET cycles consisting of at least a total of four cleavage-stage embryos or two blastocysts of good quality. Cleavage-staged embryos were graded on a scale of 0–5 based on (1) the rate of cellular division, (2) uniformity of the size of blastomeres, (3) uniformity of the shape of blastomeres, (4) clarity of the cytoplasm of blastomeres and (5) the presence of nuclear fragmentation with Grades 3–5 being usable embryos. Blastocysts were graded based on the appearance of the inner cell mass and the trophectoderm. Inner-cell-mass was graded based on how tightly packed or loosely packed are the cells, and whether there is a good layer of trophectoderm in the blastocyst, with a score of A to C being usable, and D being discarded. Inclusion criteria were women ≤40 years of age with primary subfertility with good ovarian reserve, good response to ovarian stimulation and optimal luteal phase endometrial thickness > 7 mm, normal hormonal profile and who met the clinical definition of RIF as failure to achieve a clinical pregnancy after a transfer of a minimum of four good-quality embryos in a minimum of two IVF cycles. Exclusion criteria were women > 40 years old, BMI > 35 and women who did not meet our criteria for RIF.

### Procedure

All patients underwent local endometrial injury in the mid-luteal phase (7–9 days after ovulation) for two consecutive menstrual cycles (Fig. 1) (n = 26). The procedure was performed in a standardized manner, using a Pipelle^®^ catheter (Laboratoire CCD, France) in the ambulatory setting. The catheter was introduced through the cervix and advanced towards the uterine fundus. The piston was drawn back and sheath rotated and moved back and forth within the uterine cavity to obtain the endometrial tissue. The tissue was stored in bench medium (5% newborn calf serum (Sigma-Aldrich, USA), 1% antibiotic/ antimycotic (Gibco, USA) in DMEM/F12 (Gibco) overnight and processed within 16 h of sampling. Subsequently, women underwent further fresh or frozen embryo transfer cycles as per prevailing clinical protocol at KK IVF Centre, KK Women’s and Children’s Hospital, Singapore (Fig. 1).

### Dissociation of endometrial tissue

Endometrial samples were digested for 60 min at 37 °C with 5 mg/mL of collagenase type I (Worthington, USA), 4 mg/mL of DNase 1 (Worthington), and 500 mM glucose (Sinopharm, China), with intermittent trituration every 20 min. The cell suspension was passed through a 40um cell strainer (BD Biosciences), washed in bench medium comprising 5% newborn calf serum (Sigma-Aldrich), 1% antibiotic/ antimycotic (Gibco) in DMEM/F12 (Gibco) before centrifuging over Ficoll^®^ Paque Plus (GE Healthcare) at 460 g, 15 min, 20 °C with brakes off. The mononuclear cells were collected, washed with bench medium before suspension in 2% fetal bovine serum (BSA, Sigma-Aldrich), in PBS and counted.

### Flow cytometry

Isolated cells were incubated with blocking solution (20% mouse serum (Sigma-Aldrich), 5% BSA, 2 mM EDTA (1st Base) in PBS) for 60 min before staining with SUSD2-PE (Biolegend, USA), CD45-FITC (Miltenyi, USA) for 15 min at room temperature. The cells were then washed and resuspended in staining buffer (0.5% BSA, 2 mM EDTA in PBS), and analyzed on CytoFlex flow cytometer (Beckman Coulter) using CytExpert software. Unlabeled cells and the respective isotype antibodies at the same concentration as primary antibodies (Invitrogen and Miltenyi) were used as controls.

### Magnetic bead separation of SUSD2^+^ cells

Isolation of human SUSD2^+^ cells was performed following manufacturer’s protocol using our previously published method^24^. Briefly, the whole cell population was incubated with FcR blocking reagent (Miltenyi) prior to magnetic beads sorting with MACS beads tagged with PE-flurophore on SUSD2 antibody (Miltenyi, Germany).

### Colony forming assays

SUSD2^+^ cells were plated at low densities of 50–200 cells/cm^2^ in clonogenic medium (10% FBS, 2% antibiotic/antimycotic, 1 × L-glutamine (Thermofisher, USA), of which half is changed every 3 days. Colonies formed over 3–4 weeks and were fixed and stained using 1% crystal violet solution (Sigma-Aldrich, USA) and enumerated. Cloning efficiency (%) was calculated by dividing the number of clones (> 50 cells/clone) by the number of seeded cells and multiplying by 100.

### Cell culture

SUSD2^+^ cells were plated on fibronectin (Sigma-Aldrich) coated T_25_ plates at a cell density of 1 × 10^4^ cells/cm^2^ with propagation media (1uM A83-01 (Sigma-Aldrich), 10 ng/ml bFGF (Peprotech, USA), 10 ng/ml EGF (Peprotech), 2% antibiotic/antimycotic in DMEM/F12) with media changes every three days. Cells were subcultured at confluency and the doubling time calculated as duration*ln(2)/ln (final concentration/initial concentration).

### ELISA assays

SUSD2^+^ cells were grown in propagation medium till confluency before switching to decidualization medium (2% charcoal stripped FBS (Gibco, Mexico), 0.5 mM 8-bromoadenosine-cAMP (Sigma-Aldrich), 10^−6^ M medroxyprogesterone acetate (Sigma-Aldrich), 1% antibiotic/antimycotic in DMEM/F12), which was changed every 48 h. At Day 7, the medium was collected and prolactin levels were measured using the ELISA assay (R&D Systems, USA), and IGFBP-1 semi-quantified with the DuoSet Development Kit (R&D Systems, USA), following manufacturer’s instructions.

### Reverse transcription

One ml of Trizol reagent (Invitrogen, USA) was added to each sample. 100ul of chloroform was added into each tube, the samples vortexed, and centrifuged at 4 °C for 15 min. The aqueous phase was transferred to a fresh 1.5 ml tube containing an equal volume of 70% ethanol. The solution was placed onto a RNeasy column (Qiagen, Germany) and RNA extracted as per manufacturer’s instruction. Synthesis of cDNA from 200 ng of RNA per sample was performed using the Sensiscript RT kit (Qiagen) according to manufacturer’s instructions.

### Real-time polymerase chain reaction

Real time polymerase chain reaction (PCR) were performed in triplicate, in 20 ul: 5 μl cDNA, 10 μl PowerUp™ SYBR™ Green Master Mix (ThermoFisher Scientific, NJ, USA), and 1 μl primer working solution. Thermal cycle conditions were 50 °C for 2 min, 96 °C for 2 min, then 40 cycles at 96 °C for 15 s and 60 °C for 60 s. Amplifications were monitored with the Applied Biosystems 7500 Fast Real-Time PCR System (ThermoFisher). Results were normalized against the housekeeping gene β-actin III, and relative gene expression was analyzed with the 2^−ddCt^ method. Primers used: β-actin (5′TGACGGGGTCACCCACACTGTGCCCATCTA′3 and 5′CTAGAAGCATTTGCGGTGGACGATGGAGGG′3), Prolactin^56^ (5′AAGCTGTAGAGATTGAGGAGCAAAC′3 and 5′TCAGGATGAACCTGGCTGACTA′3), and IGFBP-1^56^ (5′CGAAGGCTCTCCATGTCACCA′3 and 5′TGTCTCCTGTGCCTTGGCTAAAC′3).

### Statistical analysis

The data is presented as mean ± standard deviation when Gaussian distribution is followed and median (range) where otherwise. The proportion of SUSD2^+^ cells as well as proliferation at first and second LEI were analysed using non-parametric Mann–Whitney for unpaired or Wilcoxon signed-ranked test for paired data. Cloning efficiency, ELISA and qPCR measurements were analysed using paired Student’s t-tests. Statistical analysis was performed using Graphpad Prism 6.0. A *p* value of > 0.05 was considered as statistically significant.

## Supplementary Information

Supplementary Information.

## Data Availability

The authors confirm that the data supporting the findings are available within the article. Please contact the corresponding author for more information.
