# Supplementary material for: Subtle changes in perivascular endometrial mesenchymal stem cells after local endometrial injury in recurrent implantation failure
Source: Sci Rep. 2023 Jan 5;13:225. doi: 10.1038/s41598-023-27388-8 (PMC9816312; doi:10.1038/s41598-023-27388-8)
Supplement: Supplementary file 1 — Supplementary Information. [file 41598_2023_27388_MOESM1_ESM.pdf]

Supplementary Table 1. Detailed Embryo Transfer Cycle Data of the Patients

Patient	Embryos before LEI	Number of ET for LEI	Grade of embryos for LEI	Outcome	Experiments Involved
1	Cleavage x 3	D3 x 2	G4 and G4	Biochemical	S/C
2	Cleavage x 2 BL x 1	BL x 2	FE and FE (collapsed)	Nil	S
3	BL x 4	D3 x 2	G4 and G4	Biochemical	C
4	BL x 2	D5 x 2	Morula and EC	LB	S/C
5	BL x 2	D3 x 1	G3	Nil	S
6	Cleavage x 2 BL x 2	BL x 2	VEC and VEE	LB DCDA Twins	S/C
7	Cleavage x 4	D2 x 2	G2 and G3	Nil	C
8	Cleavage x 4	D4 x 2	G2 and Morula	LB	S
9	BL x 4	D3 x 1	G3	Nil	S
10	Cleavage x 4	BL x 1	FE (Expanding)	Nil	S
11	BL x 2	Drop Out	Drop Out		S
12	Cleavage x 4	BL x 1	FEC	Biochemical	S/C/De
13	BL x 2	BL x 1	FEB	LB	S/C/De
14	Cleavage x 4	BL x 1	FE (Expanding)	LB	S
15	Cleavage x 4	ET x 2	G4 and Morula	Nil	S/C
16	Cleavage x 4	D2 x 2	G2 and G3	Biochemical	S/C
17	Cleavage x 4 BL x 1	D3 x 2	Morula x 2	Nil	S/C
18	Cleavage x 3 BL x 1	Drop Out	Drop Out		C
19	BL x 2	BL x 1	FEC	Nil	S/C
20	Cleavage x 2 BL x 1	BL x 2	FEC and FE (Expanding)	Nil	S/C
21	BL x 2	BL x 2	PEC and PEC	Biochemical	S
22	Cleavage x 1 BL x 2	D3 x 2	G4 and G4	Nil	S/De
23	BL x 2	BL x 2	PFEC and FE (Expanding)	LB	S/De
24	BL x 3	BL x 2	FEB and FEB	LB Twins	S
25	Cleavage x 2 BL x 3	D2 x 2	G3 and G4	LB	S
26	BL x 3	BL x 2 (Hatched)	Hatched x 2	LB	S/De

Legends: For Grade of Embryos and Blastocysts: Blastocyst (BL), Embryo Day (D), Grade (G), Fully Expanded Grade B (FEB), Fully Expanded Grade C (FEC), Very Early Expanding (VEE), Very Early Cavitation (VEC), Early Cavitation (EC), Fully Expanded (FE), Pseudo Expanded Grade C (PEC), Pseudo Fully Expanded Grade C (PFEC), For others: Embryo Transfer (ET), Local Endometrial Injury (LEI), Dichorionic Diamniotic (DCDA), live birth (LB), Proportion of SUSD2 (S), Clonogenicity Assay (C), Decidualisation Assay (De).
